# Eviction, Collective Efficacy, and Firearm Violence in Chicago

**DOI:** 10.1001/jamanetworkopen.2025.49950

**Published:** 2025-12-23

**Authors:** Thomas Statchen, Anna Volerman, Louise C. Hawkley, Elizabeth L. Tung

**Affiliations:** 1 Pritzker School of Medicine, University of Chicago, Chicago, Illinois; 2 Departments of Medicine and Pediatrics, Biological Sciences Division, University of Chicago, Chicago, Illinois; 3 University of Chicago, Chicago, Illinois; 4 Department of Medicine, Biological Sciences Division, University of Chicago, Chicago, Illinois

## Abstract

**Importance:**

Firearm violence is concentrated in structurally marginalized communities. Collective efficacy—a community’s belief in their ability to achieve a shared goal—has been associated with lower rates of firearm violence. It remains unclear whether structural determinants, such as eviction, may be associated with lower collective efficacy and firearm violence.

**Objective:**

To understand if eviction is independently associated with firearm violence and if eviction moderates established associations between collective efficacy and firearm violence.

**Design, Setting, and Participants:**

This cross-sectional study used participant data from the Healthy Chicago Surveys (HCS) from 2021 to 2023, and neighborhood-level data from the City of Chicago. Participants included respondents to the HCS, and data were analyzed from July to December 2024.

**Exposures:**

Primary exposures included personal experience of eviction (self-reported) and neighborhood-level exposure to eviction (census-tract eviction rate). Neighborhood collective efficacy and related neighborhood measures were also examined in moderation analyses, modeling an interaction term between eviction and neighborhood measures.

**Main Outcome and Measures:**

The primary outcome was exposure to firearm violence, measured by shooting events within 1000 feet of a participant’s home. Mixed-effects linear regression models were used to examine firearm violence as a function of eviction measures and theoretically-relevant covariates.

**Results:**

The sample included 13 916 participants. Most participants were White (5194 [37.7%]), Black (3915 [28.4%]), or Hispanic (3150 [22.9%]); women (8625 [62.5%]) were oversampled relative to men (4923 [35.7%]). Additionally, 3362 individuals (25.2%) lived below the federal poverty line and 7032 (50.8%) had at least a bachelor’s degree. Most participants were age 45 to 64 years (4416 [31.8%]) or 30 to 44 years (4169 [30.0%]). The median (IQR) number of shootings within 1000 feet of a participant’s home was 3 (1-9). Each percentage increase in census tract eviction rate (mean [range], 0.88% [0%-5.33%]) was associated with 2.66 (95% CI, 2.01-3.31) additional shootings within 1000 ft of the participant’s home. Individual experience of eviction was associated with 1.04 (95% CI, 0.46-1.61) additional shootings within 1000 ft. Eviction was a significant moderator of associations of low collective efficacy with firearm violence (0.89; 95% CI, 0.20 to 1.58; *P* = .01).

**Conclusions and Relevance:**

In this cross-sectional study, eviction was associated with increased firearm violence exposure. Eviction augmented associations between low collective efficacy and firearm violence, highlighting the contextual role of structural disadvantage in galvanizing this association. Eviction may be a tangible intervention target for violence prevention in US cities.

## Key Points

**Question:**

What is the association of eviction with firearm violence in Chicago, Illinois, and how does eviction moderate the association between collective efficacy and firearm violence?

**Findings:**

In this cross-sectional study of 13 916 Chicago residents, a 1% increase in the census tract eviction rate was associated with 2.66 additional shootings within 1000 feet of the individual’s home. Eviction was also a significant moderator of collective efficacy—a community’s belief in their ability to achieve a shared goal—augmenting associations of low collective efficacy with firearm violence.

**Meaning:**

These findings suggest that eviction is a possible structural disadvantage associated with firearm violence in Chicago.

## Teaser

This cross-sectional study examines whether eviction rates are associated with firearm violence in Chicago and whether eviction moderates the established association between collective efficacy and firearm violence.

## Introduction

Firearm violence is a major challenge in the US, particularly in urban neighborhoods with concentrated socioeconomic disadvantages. In a study of 5 major US cities, more than 55% of shootings occurred in just 9% of census tracts, with small increases in socioeconomic disadvantage associated with large increases in firearm violence.^1^ In Chicago, firearm violence is concentrated on the south and west sides, which include racially minoritized neighborhoods with a history of structural disinvestment resulting in residential segregation, economic exclusion, and stark gaps in life expectancy.^2,3^ Despite reductions in firearm violence in Chicago from 2004 to 2014, the cumulative risk of firearm injury for young people, the segment of the population at highest risk, has changed very little over the last 20 years because of surges in violence in 2016 and 2020.^4^

Due to the concentration of firearm violence within specific neighborhoods, there has been sustained interest in identifying neighborhood-level characteristics associated with firearm violence.^5^ One prominently studied characteristic, first identified by Sampson et al,^6^ is collective efficacy—residents’ shared belief in their ability to work together on behalf of the common good. Sampson et al^6^ found that this was a key factor in promoting neighborhood safety, while low collective efficacy was associated with higher rates of violent crime. It has been theorized that in neighborhoods with high collective efficacy, residents enact informal social controls, including monitoring residents’ behaviors, limiting social disorder, and supporting collective responses to shape public funding and policies that impact their community.^6^ These collective actions improve safety and reduce interpersonal violence—a shared goal for members of a community. The association between low collective efficacy and neighborhood violence has been consistently demonstrated elsewhere, including Indianapolis, Indiana; Mesa, Arizona; and additional studies in Chicago.^7,8,9^

We expand on collective efficacy to include the study of related neighborhood social dimensions, such as belonging and social capital. Belonging is defined as the degree to which people feel they are part of their neighborhood. Social capital is defined as the networks of people and relationships from whom someone could ask for help if they needed it. Both have been identified in prior literature as distinct constructs from collective efficacy, with social capital being correlated with rates of collective efficacy and belonging being more distinct.^10^ Each of these neighborhood social measures is the product of intersecting factors, including structural processes, such as economic disinvestment and social marginalization.^11,12,13^ Thus, it is necessary to understand upstream structural factors that influence these social processes to provide policymakers and communities with intervention footholds.

One such structural factor is eviction, which is the forcible removal of a tenant from their housing. While eviction is a marker of financial strain and instability in a community, it can also reflect policies and structural processes that govern how poverty is managed. In the US annually between 2007 and 2016, 7 600 000 people faced the threat of eviction, with 3 600 000 people evicted.^14,15,16^ There are significant racial disparities in who is impacted, with Black women disproportionately targeted by eviction filings.^17^ Eviction has important impacts at both the individual and community levels. People who are evicted are more likely to experience material hardship, depression, and stress.^17^ On a community level, high eviction rates correspond with lower voter turnout, fewer city service calls to report neighborhood issues, and increased maternal mortality.^17,18,19^ Eviction, or the threat of eviction, can disrupt stability for both the affected individual and their neighbors, which may be expected to worsen collective efficacy and, therefore, reduce constraints on firearm violence.^20^ Concurrently, eviction—as a structural disadvantage—may create an adverse neighborhood context within which collective efficacy becomes critical. Some studies have begun to explore the association between eviction and firearm violence, demonstrating a positive correlation between increased rates of eviction and violent crime in Boston and Philadelphia.^21,22,23^ However, to our knowledge, no studies have specifically examined how eviction and collective efficacy may interact in their association with firearm violence in neighborhoods.

The purpose of this study was to examine the associations between firearm violence, eviction, and collective efficacy. We analyzed data from the City of Chicago to examine: (1) whether a resident’s exposures to both individual- and neighborhood-level evictions were associated with higher rates of firearm violence near their home; and (2) whether eviction functioned as a moderator of established associations between neighborhood collective efficacy and firearm violence. We theorized that eviction could destabilize neighborhood collective efficacy and, therefore, hypothesized that eviction would be associated with higher rates of firearm violence. We also theorized that eviction is a structural disadvantage that changes the context of a neighborhood and may augment associations between low collective efficacy and firearm violence. Alternatively, collective efficacy may be less important in neighborhoods with fewer structural disadvantages.

## Methods

This study was conducted using pooled cross-sectional survey data from the Healthy Chicago Survey (HCS), an annual survey conducted by the Chicago Department of Public Health. The HCS uses address-based sampling to produce representative estimates for each of Chicago’s 77 community areas.^24^ Data were drawn from surveys collected annually between August and December from 2021 to 2023. Earlier HCS data were not used because of method changes that prohibited pooling across earlier time periods. Individual-level HCS data were paired with publicly available data on evictions and firearm violence from the City of Chicago. This cross-sectional study was approved with a waiver of informed consent by the University of Chicago institutional review board. This research followed the Strengthening the Reporting of Observational Studies in Epidemiology (STROBE) reporting guideline.

### Main Measures

The primary dependent measure was individual-level exposure to firearm violence among HCS participants. The home address of each participant was geocoded and paired with a publicly available dataset of firearm shooting events published by the Chicago Police Department. The number of shootings was calculated within 1000-feet radii of each participant’s home address over the study period, with sensitivity testing at 500- and 1500-feet radii. The number of shootings within each radius (ie, effective shooting density) was used as a proxy for exposure to firearm violence, with the understanding that nearby shootings impact residents even if residents are not home when shootings occur (eg, observing yellow caution tape where a shooting occurred for many days). This measure was selected to capture the full burden of firearm violence, compared with measures like the number of homicides, which has fewer total events and does not account for the psychological and physical impacts of nonfatal shootings.

Primary independent measures included exposure to eviction, collective efficacy, and related measures of belonging and social capital. Exposure to eviction was examined at the individual and census tract levels. Individual-level eviction was self-reported in the HCS. Participants were asked: “Since the start of the COVID-19 pandemic in March 2020, have you been evicted or forced to move?” Census tract-level eviction rates were measured using data from a 2018 Cook County Sheriff’s Office dataset, which reflects the most recent information available. The eviction rate was analyzed as the percentage of renter-occupied housing units that experienced an eviction in the previous year.

All neighborhood social measure responses were converted to a tract-level variable for analysis and modeled as a neighborhood effect. Collective efficacy was measured with a 4-point Likert scale question, “To what extent do you and your neighbors have the ability to impact your community?” Neighborhood belonging was measured with a 5-point Likert scale question, “Would you say that you really feel a part of your neighborhood?” Social capital was measured using numeric input to the question, “About how many people in your community do you know well enough to ask for help if you need it?” To determine a census tract-level measure, the mean level of each characteristic within a census tract was calculated.

Covariates were selected using both individual-level demographic characteristics from the HCS and census tract-level characteristics from the 2022 American Community Survey (ACS). Individual-level, self-reported characteristics included poverty level (less than 100%, 100% to 200%, 200% to 300%, 400% or more), race or ethnicity (Asian, Black, Hispanic, White, other), gender (cis man, cis woman, or trans, nonbinary, other), age (18 to 24 years, 25 to 29 years, 30 to 44 years, 45 to 64 years, and 65 years or older), number of people living in the household (continuous), and level of education (less than high school, high school, some college or associate’s degree, bachelor’s degree or higher). The racial category of other included individuals who identified as American Indian or Alaska Native, Native Hawaiian or Pacific Islander, or some other race in the HCS. Census tract-level characteristics included the percentage of people living in poverty, the percentage of people with less than a college degree, the percentage of people who had moved in the past year, and the median length of time lived in the census tract. The median length of time lived was included to indicate overall mobility in the census tract, isolating the association of forced moves through eviction. The percentage of people identifying as non-Hispanic Black and Hispanic was also considered but not included in the final models due to multicollinearity with individual-level race or ethnicity.

### Statistical Analysis

Descriptive statistics were calculated for all individual- and census tract-level demographic characteristics. In primary analyses, mixed-effects linear regression models were used to examine associations between eviction, both individual- and census tract-level, and individual-level exposure to firearm violence at each radius (ie, shooting events per address-based radius). The number of shooting events was modeled as a function of both eviction measures, with adjustment for all covariates. For each model, individual HCS participants were nested within census tracts.

In secondary analyses, the number of shooting events was modeled as a function of the interaction between census tract-level eviction rates and census tract-level measures of collective efficacy, belonging, and social capital, with adjustment for all aforementioned covariates. In both analyses, *P* < .05 was used as the threshold for statistical significance. Two-sided tests were used to determine significance. Data analysis was conducted in R version 2024.04.2 (R Project for Statistical Computing) using the packages sf version 1.0.16, lme4 version 1.1.35.5, and interactions version 1.2.0. Data were analyzed from July to December 2024.

## Results

Between 2021 and 2023, the HCS received 13 916 responses (8628 women [62.5%], 4923 men [35.7%]; 884 Asian [6.4%], 3915 Black [28.4%], 3150 Hispanic [22.9%], 5194 White [37.7%]). Additionally, 3362 participants (25.2%) were below the federal poverty line, while 5199 (38.9%) were over 400% of the federal poverty line; 7032 individuals (50.8%) had a college degree. In total, 325 survey participants (2.3%) reported personal experience of eviction or a forced move since March 2020 (beginning of the COVID-19 pandemic). The study population characteristics are shown in Table 1.

**Table 1.** Characteristics of Respondents to Healthy Chicago Survey, Chicago, 2021-2023

Characteristic (n = 13 916)	Participants, No. (%)
Race and ethnicity	
Asian	884 (6.4)
Black	3915 (28.4)
Hispanic	3150 (22.9)
White	5194 (37.7)
Other^b^	628 (4.6)
Gender	
Cis man	4923 (35.7)
Cis woman	8628 (62.5)
Trans, nonbinary, or other	249 (1.8)
Poverty level, % FPL	
<100	3362 (25.2)
100-199	2006 (15.0)
200-399	2788 (20.9)
≥400	5199 (38.9)
Education	
Less than high school	885 (6.4)
High school or GED	2191 (15.8)
Some college or associate’s	3747 (27.0)
Bachelor’s or higher	7032 (50.8)
Age, y	
≥65	3052 (22.0)
45-64	4416 (31.8)
30-44	4169 (30.0)
25-29	1421 (10.2)
18-24	834 (6.0)
No. living in household	
1	5100 (37)
2	3918 (28)
3-4	3360 (24)
5 or more	1401 (10)
Past eviction	
Evicted since 2020	325 (2.3)
Not evicted since 2020	13 591 (97.7)
Extent you and your neighbors can impact community (collective efficacy)	
Great extent	2037 (14.7)
Somewhat	5465 (39.4)
A little	4109 (29.6)
Not at all	2273 (16.4)
Really feel a part of your neighborhood (belonging)	
Strongly agree	1429 (10.3)
Agree	4615 (33.2)
Neither agree or disagree	5512 (39.7)
Disagree	1543 (11.1)
Strongly disagree	789 (5.7)
No. of people you could ask for help (social capital), median (IQR)	3.0 (1.0-5.0)

Abbreviations: FPL, federal poverty level; GED, General Educational Development (alternative to high school diploma).

^a^ Racial category of other included individuals responding as American Indian or Alaska Native, Native Hawaiian or Pacific Islander, or some other race in the Healthy Chicago Survey.

The mean (range) eviction rate was 0.88% (0%-5.33%) across census tracts. Eviction was most concentrated on Chicago’s south and west sides. The median (IQR) number of shootings within 1000 feet of a participant’s home was 3 (1-9) shootings. High shooting rates were also concentrated in specific census tracts on the south and west sides of Chicago.

Collective efficacy was evenly distributed within the sample. Nearly half (6382 [45.9%]) of participants indicated feeling a low degree of collective efficacy. Fewer individuals indicated a low degree of belonging in their community—2332 participants (16.8%) indicated a low degree of belonging in their neighborhood, while 5512 (39.7%) indicated a neutral feeling of belonging. For social capital, participants reported a median (IQR) of 3 (1-5) people they could ask for help.

Increased exposure to eviction was consistently associated with an increased number of shootings occurring near an individual’s home (Table 2 and eTable in Supplement 1). Each percentage increase in the eviction rate in a census tract was associated with 2.66 (95% CI, 2.01-3.31) more shootings within 1000 ft of a participant’s home (*P* < .001). Findings were consistent at 500 ft and 1500 ft sensitivity thresholds (Table 2). Similarly, personal experience of eviction was associated with 1.04 (95% CI, 0.46-1.61) more shootings within 1000 ft of a participant’s home (*P* < .001), with consistent findings at 500 ft and 1500 ft. This finding was the largest individual-level association with shootings near the home, greater in magnitude than Black race (0.72 more shootings), having an income more than 400% of the federal poverty line (0.66 less shootings), or having less than a high school diploma (0.79 more shootings).

**Table 2.** Associations of Eviction Exposure With Firearm Violence Exposure, Chicago, 2021-2023

Characteristics^a^	Shootings within 500 ft		Shootings within 1000 ft		Shootings within 1500 ft	
	β (95% CI)	*P* value	β (95% CI)	*P* value	β (95% CI)	*P* value
Renter-occupied households evicted in 2018 (%)	0.61 (0.40-0.81)	<.001	2.66 (2.01-3.31)	<.001	5.96 (4.67-7.26)	<.001
Past eviction	0.33 (0.07-0.60)	.015	1.04 (0.46-1.61)	<.001	0.96 (0.10-1.82)	.03

^a^ See eTable in Supplement 1 for full model with covariates. Both individual- and community-level eviction were included in the same multivariable model for each shooting radius.

In secondary analyses, low collective efficacy was associated with firearm violence. Eviction was a significant moderator of associations of low collective efficacy (0.89; 95% CI, 0.20 to 1.58; *P* = .01), belonging (1.13; 95% CI, 0.40 to 1.87; *P* = .003), and social capital (1.08; 95% CI, 0.29 to 1.87; *P* = .008) with firearm violence (Figure, each scaled, range 0-5). In upper-quartile eviction rate census tracts, a 1-unit decrease in average perception of collective efficacy was associated with 1.23 (95% CI, 0.40 to 2.07) more shootings within 1000 ft of residents (*P* = .004). By contrast, in lower quartile eviction rate census tracts, a 1-unit decrease in collective efficacy was associated with 0.39 (95% CI, −0.67 to 1.45) more shootings (*P* = .47). An association between social capital and shootings was only present in the upper quartile (0.93; 95% CI, 0.03 to 1.84; *P* = .04) and 90th percentile eviction rate census tracts (1.92; 95% CI, 0.70 to 3.15; *P* = .002), with a null association in median- and lower quartile-eviction census tracts. An association between belonging and shootings was present in all eviction rate stratified groups, but the association was stronger in the upper quartile and 90th percentile groups (90th percentile eviction rate: 3.36; 95% CI, 2.18 to 4.54; *P* < .001; upper quartile eviction rate: 2.32; 95% CI, 1.38 to 3.26; *P* < .001; median eviction rate: 1.59; 95% CI, 0.55 to 2.63; *P* = .003; lower quartile eviction rate: 1.25; 95% CI, 0.10 to 2.40; *P* = .03).

**Figure.**
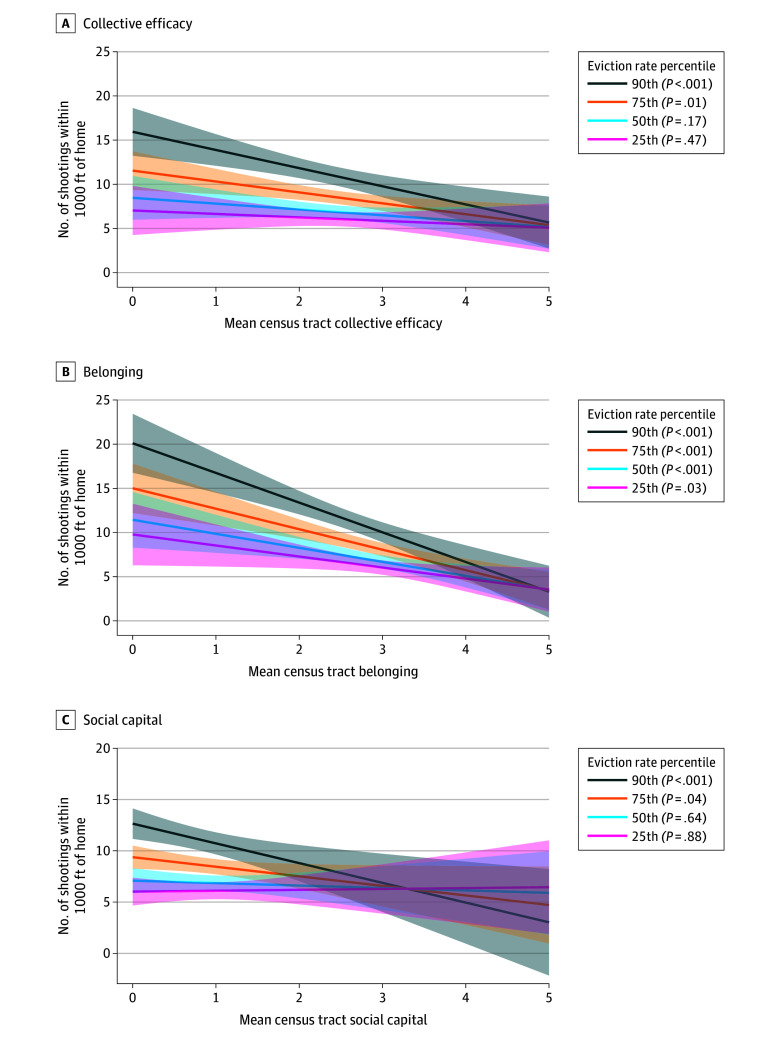
Moderating Effect of Eviction on Social Characteristics Association With Firearm Violence Associations of collective efficacy (A), belonging (B), and social capital (C) with firearm violence, stratified by eviction rate percentile, Chicago, 2021-2023. The shading indicates 95% CI.

## Discussion

In this Chicago-based study, we found higher exposure to firearm violence among residents personally exposed to eviction or living in census tracts with higher eviction rates. Our study is the first to demonstrate this association using both individual- and census tract-level eviction, as well as individual-level exposure to firearm violence, an association that remains significant even after controlling for other measures of neighborhood disadvantage, such as residential mobility and poverty. These findings extend prior research, which has demonstrated associations between community-level eviction and crime.^22^

We found that both personal and census-tract evictions were associated with shootings within a radius of an individual’s home. Census tract-level eviction rate remained significant even when controlling for overall community mobility, emphasizing the importance of eviction as a forced, unplanned move. Census tract-level eviction rates had stronger associations with firearm violence than personal experience of eviction, although both were significant. This finding likely reflected the importance of neighborhood-level processes in understanding the role of eviction in communities. Personal exposure to eviction can have devastating impacts on an individual’s life, but high eviction rates impact the social fabric of a community. These results corroborate prior literature documenting adverse consequences of eviction, including reduced engagement with the health care system,^25^ impaired childhood development leading to lower cognitive scores,^26^ and persistent material hardship.^27^

Importantly, we add to the existing literature by demonstrating that eviction moderated associations between low collective efficacy and firearm violence. The associations of low collective efficacy, social capital, and belonging with firearm violence were strongest in high eviction census tracts. We theorize that eviction—a key structural disadvantage impacting neighborhoods—may create a unique context for the importance of collective efficacy in mitigating firearm violence. While past studies have shown a strong association between low collective efficacy and firearm violence, our work suggests that the presence of structural disadvantage is key in this association. In a context where there was low or no eviction, the level of collective efficacy was not associated with firearm violence. This finding raises the possibility that the level of collective efficacy may not be associated with violence in all contexts, but rather, may mitigate the consequences of structural disadvantage in certain contexts. We theorize that collective efficacy may in fact mask the deleterious associations of structural disadvantage with firearm violence in neighborhoods with strong communities, while it appears to be less important in neighborhoods with fewer structural disadvantages.

Our findings have important policy implications. Collective efficacy and related neighborhood measures have been identified as playing an important role in firearm violence but are difficult to intervene on. Eviction is a clearer intervention target that moderates low collective efficacy and its association with firearm violence. Eviction moratoriums, rent stabilizing measures, and additional resources for households at risk of eviction are all policy opportunities through which residential stability and reduced violence in communities could be achieved.^21,27^ For example, subsidized, multifamily housing in Atlanta was shown to have a significantly lower eviction rate than market-rate units.^28^ Furthermore, broad COVID-19 pandemic-related policies, including eviction moratoria and renter-supportive measures, led to reduced eviction filings in 31 cities.^29^ Re-implementing protections for renters or changing zoning policy to support the construction of affordable housing in cities could have significant impacts on community health and vitality, and thus have implications for firearm violence.

### Limitations

This study has limitations. First, we used pooled cross-sectional data, and causal relationships cannot be determined. Although we theorized that eviction may increase firearm violence, it is also possible that firearm violence increases eviction. Furthermore, the study included data collected over a 3-year time period. While this improves the precision of pooled estimates, it does not account for variation within the study period. Second, collective efficacy and related measures were sampled among adults and may not reflect the perspectives of younger age groups who have high exposure to community violence. Future studies should examine age groups younger than 18 years old. Third, the original HCS data were sampled at the community area level, rather than the census tract level. As such, sampling weights were not used, and findings should be interpreted as nonrepresentative of Chicago. However, the sample demographics were similar to the city’s demographics. Finally, measures of neighborhood social processes may be less sensitive than previous studies, because the HCS included only 1 survey item for each construct (3 items total). By contrast, Sampson et al^6^ used a 5-item measure to determine collective efficacy.

## Conclusions

In this study, we found that exposure to eviction at the individual- and census tract-levels were associated with higher exposure to firearm violence. Importantly, our findings suggest that eviction, as a form of structural disadvantage, moderates long-established associations between collective efficacy and firearm violence. Thus, eviction creates a neighborhood context within which collective efficacy matters. Our findings highlight the importance of policies to reduce eviction or mitigate other structural disadvantages in conjunction with social and behavioral interventions to combat firearm violence. Future research should seek to establish a causal association between eviction and firearm violence. More comprehensive data collection about evictions could permit longitudinal analysis of the impact of these policies on eviction rates, firearm violence, and neighborhood social characteristics. These findings suggest that eviction may be a policy foothold for combatting structural inequities driving cycles of residential instability and firearm violence within neighborhoods.

## Notes

Supplement 1. **eTable.** Associations of Eviction Exposure With Firearm Violence Exposure, Chicago, 2021 to 2023

Supplement 2.
Data Sharing Statement
