# Supplementary material for: Eviction, Collective Efficacy, and Firearm Violence in Chicago
Source: JAMA Netw Open. 2025 Dec 23;8(12):e2549950. doi: 10.1001/jamanetworkopen.2025.49950 (PMC12728654; doi:10.1001/jamanetworkopen.2025.49950)
Supplement: Supplement 1. — eTable. Associations of Eviction Exposure With Firearm Violence Exposure, Chicago, 2021 to 2023 [file jamanetwopen-e2549950-s001.pdf]

Supplemental Online Content

Statchen T, Volerman A, Hawkley LC, Tung EL. Eviction, collective efficacy, and firearm violence in Chicago. *JAMA Netw Open*. 2025;8(12):e2549950.
doi:10.1001/jamanetworkopen.2025.49950

eTable. Associations of Eviction Exposure With Firearm Violence Exposure, Chicago, 2021-2023

This supplemental material has been provided by the authors to give readers additional information about their work.

© 2025 Statchen T et al. *JAMA Network Open*.

**eTable.** Associations of Eviction Exposure With Firearm Violence Exposure, Chicago, 2021-2023									
	Shootings within 500ft			Shootings within 1000ft			Shootings within 1500ft		
**Characteristic**	**Beta**	**95% CI**	**p-value**	**Beta**	**95% CI**	**p-value**	**Beta**	**95% CI**	**p-value**
**Renter-Occupied Households Evicted in 2018 (%)**	0.61	0.40, 0.81	<0.001	2.66	2.01, 3.31	<0.001	5.96	4.67, 7.26	<0.001
**Past Eviction**	0.33	0.07, 0.60	0.015	1.04	0.46, 1.61	<0.001	0.96	0.10, 1.82	0.028
**Poverty Level**									
**<100% FPL**	—	—		—	—		—	—	
**100-199% FPL**	-0.11	-0.25, 0.03	0.12	-0.36	-0.65, -0.07	0.016	-0.63	-1.07, -0.20	0.004
**200-399% FPL**	-0.16	-0.29, -0.03	0.019	-0.44	-0.72, -0.16	0.002	-0.76	-1.17, -0.35	<0.001
**400+% FPL**	-0.11	-0.24, 0.02	0.1	-0.37	-0.66, -0.09	0.01	-0.68	-1.10, -0.26	0.002
**Race/Ethnicity**									
**White**	—	—		—	—		—	—	
**Black**	0.47	0.31, 0.63	<0.001	0.73	0.38, 1.08	<0.001	0.81	0.29, 1.33	0.002
**Asian**	-0.01	-0.19, 0.17	>0.9	0.04	-0.35, 0.43	0.9	-0.07	-0.65, 0.51	0.8
**Other**	0.02	-0.19, 0.23	0.9	0.17	-0.28, 0.62	0.5	0.04	-0.64, 0.71	>0.9
**Hispanic**	-0.02	-0.16, 0.11	0.7	-0.09	-0.38, 0.20	0.5	-0.07	-0.50, 0.35	0.7
**Gender**									
**Cis Man**	—	—		—	—		—	—	
**Cis Woman**	-0.02	-0.11, 0.07	0.6	0.16	-0.02, 0.35	0.085	0.17	-0.10, 0.45	0.2
**Trans/Non-binary/Other**	0.09	-0.22, 0.41	0.6	-0.27	-0.94, 0.41	0.4	-0.45	-1.45, 0.55	0.4
**Education**									
**Less than High School**	—	—		—	—		—	—	
**High School or GED**	-0.25	-0.45, -0.05	0.014	-0.79	-1.22, -0.36	<0.001	-0.58	-1.22, 0.06	0.074
**Some College/Associates**	-0.24	-0.43, -0.05	0.013	-0.56	-0.97, -0.14	0.008	-0.36	-0.98, 0.25	0.2
**Bachelor's or Higher**	-0.36	-0.56, -0.16	<0.001	-0.75	-1.18, -0.33	<0.001	-0.54	-1.17, 0.09	0.1
**Age**									
**65+**	—	—		—	—		—	—	
**45-64**	0.07	-0.05, 0.19	0.3	0.2	-0.05, 0.45	0.12	0.28	-0.10, 0.65	0.15
**30-44**	0.21	0.08, 0.34	0.001	0.37	0.10, 0.64	0.008	0.55	0.15, 0.95	0.007
**25-29**	0.24	0.07, 0.40	0.005	0.64	0.29, 0.99	<0.001	0.88	0.35, 1.40	0.001
**18-24**	0.43	0.23, 0.63	<0.001	0.72	0.30, 1.14	<0.001	1.16	0.53, 1.78	<0.001
**Number Living in Household**	0.03	0.01, 0.05	0.014	0.05	0.01, 0.10	0.023	0.05	-0.02, 0.12	0.2
**Population in Poverty (%)**	0.07	0.05, 0.08	<0.001	0.23	0.18, 0.29	<0.001	0.46	0.35, 0.57	<0.001
**Population with Less than a College Degree (%)**	0.03	0.02, 0.04	<0.001	0.11	0.08, 0.14	<0.001	0.23	0.16, 0.29	<0.001
**Median Years Lived in Census Tract**	-0.06	-0.11, -0.01	0.026	-0.22	-0.38, -0.06	0.007	-0.47	-0.80, -0.15	0.004
**Population that moved in past year (%)**	-0.04	-0.06, -0.01	0.002	-0.12	-0.20, -0.04	0.003	-0.22	-0.38, -0.06	0.006

© 2025 Statchen T et al. *JAMA Network Open*.
