# Supplementary material for: Eviction, Collective Efficacy, and Firearm Violence in Chicago
Source: JAMA Netw Open. 2025 Dec 23;8(12):e2549950. doi: 10.1001/jamanetworkopen.2025.49950 (PMC12728654; doi:10.1001/jamanetworkopen.2025.49950)
Supplement: Supplement 2. — Data Sharing Statement [file jamanetwopen-e2549950-s002.pdf]

Data Sharing Statement

Statchen. Eviction, Collective Efficacy, and Firearm Violence in Chicago. *JAMA Netw Open*. Published December 23, 2025. doi:10.1001/jamanetworkopen.2025.49950

Data
Data available: No

Additional Information
Explanation for why data not available: As geocoded data from the Healthy Chicago Survey is identifiable and managed by the Chicago Department of Public Health it is not able to be made available publicly. Publicly available data used in the study is already available from the Chicago Data Portal (data.cityofchicago.org), Chicago Health Atlas (chicagohealthatlas.org), or the American Community Survey.
